# Match vs. Training Physical Requirements and Their Association with Field-Based Physical Tests in International CP Football

**DOI:** 10.3390/sports13090312

**Published:** 2025-09-08

**Authors:** Juan Francisco Maggiolo, Alejandro Caña-Pino, Manuel Moya-Ramón, Iván Peña-González

**Affiliations:** 1 Department of Sports Sciences, Sports Research Centre, Miguel Hernández University, 03202 Elche, Spainmmoya@umh.es (M.M.-R.); ipena@umh.es (I.P.-G.); 2 Surgical Medical-Therapy Department, Medicine Faculty and Health Sciences, University of Extremadura, 06006 Badajoz, Spain; 3 Research Group PhysioH (Fisioterapia e Hipoterapia), University of Extremadura, 06006 Badajoz, Spain

**Keywords:** soccer, physical demands, physical performance, para-sport, brain impairment

## Abstract

Objetives: This study aimed to (1) describe and compare the external physical requirements of international cerebral palsy (CP) football players during training sessions and official matches at the 2024 IFCPF World Cup, and (2) analyze the relationships between standardized field-based physical performance tests and the physical requirements recorded in both contexts. Methods: Twelve international outfield players from the Spanish national CP football team were monitored throughout the tournament. Physical performance was evaluated two weeks prior using 5-m and 30-m sprints, a Modified Agility Test (MAT), a dribbling test, and the 30–15 Intermittent Fitness Test (vIFT). Match and training physical requirements were assessed using inertial devices, including total and relative distances, velocity metrics, and acceleration/deceleration outputs. Results: Matches imposed significantly greater demands than training sessions in terms of peak velocity, total distance per minute, and distance at moderate (>12–18 km/h) and high (>18 km/h) intensities (t = 2.79 to 8.06; *p* = 0.01; ES(d) = 0.50 to 1.45). Training sessions exhibited greater variability in load while match requirements were consistent across games. Performance in the MAT and dribbling tests correlated with several physical indicators in both training and competition. In contrast, vIFT and sprint tests showed limited associations, especially with match variables. Conclusions: Match play elicits higher and more stable physical requirements than training. The MAT and dribbling tests appear to be ecologically valid tools for assessing functional readiness in CP football. These findings support the integration of specific physical tests and tailored training designs to better replicate the competitive requirements of international CP football.

## 1. Introduction

Cerebral palsy (CP) football is competitive team para-sport designed for athletes with neurological impairments affecting motor control, coordination, and balance. CP football is governed by the International Federation of CP Football (IFCPF), and players compete under a modified set of FIFA rules [1]. The physical demands of CP football closely resemble those of mainstream football, as it is a high-intensity intermittent sport characterized by short bursts of intense actions (e.g., sprinting or jumping) interspersed with longer periods of recovery or lower-intensity activities [2,3,4,5]. Despite the structural adaptations, CP football shares many of the physical and tactical requirements of mainstream football, including frequent high-intensity efforts, rapid accelerations and decelerations, changes of direction, and complex technical actions performed under physical and cognitive pressure. These requirements are further modulated by the nature of the impairment, which can affect intersegmental coordination, motor control, and fatigue resistance [6,7,8]. As a result, accurately quantifying physical requirements and performance capacities in this sport is essential for evidence-based training design, load management, and player development.

Although field-based physical performance assessments are widely used in CP football to monitor player readiness and guide training decisions [9,10], their ecological validity—that is, their ability to reflect the actual physical requirements of players in competition—remains under investigation. Field-based tests provide standardized measures of physical capacities [11], but their transferability to competitive performance remains under debate, as they cannot fully capture the perceptual, tactical, and contextual demands inherent to match play [12]. Recent evidence has shown that some physical performance tests are moderately to strongly associated with specific match-derived locomotor variables, particularly in international-level players [9]. Notably, linear sprint and intermittent endurance demonstrated the strongest associations with variables such as total distance covered, average velocity, and distances at moderate and high intensities [9]. These findings support the partial predictive validity of certain field-based tests in capturing game-relevant physical outputs. However, the strength and consistency of these associations appear to vary depending not only on the specific test and performance variable analyzed, but also on a range of contextual factors. These include the tactical system employed by the team, the strategic role assigned to individual players, the level and style of the opposition, and other situational variables such as match status, environmental conditions, or tournament phase [13]. As such, physical test performance should be interpreted with caution and in conjunction with contextual information when used to infer real-game requirements in CP football.

However, there is still a notable gap in the literature regarding the relationship between physical performance test outcomes and the physical requirements experienced during training sessions. While match contexts have been increasingly analyzed in recent years [3,4,14], to the best to our knowledge, no study to date has systematically examined whether performance in field-based tests reflects the locomotor and mechanical requirements encountered during CP football training, where physical load may vary substantially depending on task design, tactical focus, or tournament periodization [12]. It is plausible that players with better physical test results are more capable of reaching higher locomotor and mechanical outputs during training tasks, potentially enabling them to approach the physical thresholds required in competition. Conversely, it is also possible that the physical requirements observed in training are primarily shaped by the structure and intensity of the training tasks themselves, rather than by the players’ individual performance capacities. This uncertainty limits the ability of coaching and performance staff to evaluate the real-world transferability of performance tests to training environments, thus hindering the optimization of load planning and individualized conditioning. Accordingly, there is a pressing need to investigate whether commonly used field tests are also ecologically valid in relation to training demands, not just competitive performance, in elite para-football.

Furthermore, there is limited evidence directly contrasting the physical requirements of training sessions and official matches in CP football. In mainstream football, however, a growing body of literature indicates that competitive matches generally elicit greater external loads than training—particularly in metrics such as moderate- and high-intensity distance, peak velocity, accelerations, and decelerations [15,16,17]. For instance, Riboli et al. (2025) observed large to very large correlations between high-intensity and acceleration/deceleration volumes in training and those in matches within a top-class professional team, suggesting that well-structured training can partially replicate match requirements [18]. Similarly, youth-national team studies have shown that training volume systematically decreases closer to match day, but the relative intensity on MD-3 and MD-1 remains high, indicating tactical tapering strategies to mirror competition demands [19]. This variation in daily training load reflects intentional periodization strategies, where fluctuations across the microcycle (e.g., tapering before matches and recovery-oriented sessions immediately after) are expected and play a key role in optimizing readiness and minimizing fatigue during competitive periods. Other investigations using small-sided game (SSG) formats have demonstrated that larger pitch sizes and formats like 10 vs. 10 can elicit external load intensities comparable to match play, particularly in distance per minute and high-intensity efforts [20]. Nevertheless, detailed comparisons often reveal significantly higher metrics during official matches, especially for peak speed and sprint distance [21]. It is also important to note that, during congested competitive periods such as international tournaments, training sessions are primarily designed to facilitate recovery and optimize readiness for subsequent matches rather than to replicate the peak physical demands of competition, particularly in squads with limited roster depth [22].

In sum, while there is strong evidence in able-bodied football that match demands typically exceed training loads, certain training designs—such as targeted drills, tactical systems, or workload tapering—can narrow this gap. Whether these patterns hold true in CP football, where training must be adapted to individual functional limitations and tournament match congestions, remains unclear. Understanding this relationship is critical for optimizing training periodization and ensuring that preparation sessions for elite CP footballers sufficiently reflect the demands of competition. In this context, the present study had two main aims. First, to describe and compare the external physical requirements of international CP football players during training sessions and official matches throughout the 2024 IFCPF World Cup. Second, the study aimed to examine the relationships between players’ field-based physical performance—assessed through standardized tests—and the physical requirements recorded during matches and training sessions. By exploring these associations, we sought to determine the extent to which commonly used physical performance tests are related to the actual physical requirements of players during competitive and training contexts.

## 2. Materials and Methods

### 2.1. Design

A cross-sectional study design was employed to examine the physical performance of international cerebral palsy (CP) football players in field-based tests and its relationship with their physical requirements during the 2024 IFCPF World Cup. Physical performance was assessed through standardized field-based tests conducted two weeks prior to the start of the tournament. In parallel, physical requirements were monitored during both training sessions and official matches throughout the World Cup. All players were previously familiar with the field test protocols, which are regularly integrated into the team’s long-term performance evaluation routine. Moreover, participants were accustomed to wearing tracking devices during both training and competition, as part of the team’s established monitoring practices.

### 2.2. Participants

The participants in this study were members of the Spanish national cerebral palsy (CP) football team, ranked among the top ten teams globally. The squad consisted of 14 male players, which is the maximum number of eligible athletes permitted to compete in the IFCPF World Cup. All players held an active IFCPF license and had an average of 10 years of competitive experience in CP football. However, two goalkeepers were excluded from the analysis due to the distinct physical and positional demands associated with their role compared to outfield players. Consequently, the final sample included 12 international outfield players with CP (FT1 [severe impairment] = 1; FT2 [moderate impairment] = 10; FT3 [mild impairment] = 1), with a mean age of 26.1 ± 5.8 years, mean body mass of 68.7 ± 8.1 kg, and mean height of 174.2 ± 5.3 cm. The class distribution reflects the regulations of the IFCPF competition system, which stipulate that at least one FT1 must be on the field at all times and no more than one FT3 can play simultaneously. In this squad, the two goalkeepers (not included in the analysis) were classified as FT1, which further explains the small representation of FT1 and FT3 players in the analyzed sample. Prior to data collection, all participants were fully informed about the study objectives, procedures, and design, and provided written informed consent in accordance with the ethical standards approved by the relevant ethics committee (DCD.IPG.060523).

### 2.3. Procedures

#### 2.3.1. Physical Fitness Assessment

Prior to the start of the 2024 IFCPF World Cup, a physical performance assessment was carried out two weeks before the tournament began. During a single testing session, participants’ body mass and height were measured using a digital scale (Tanita BC 601 Ltd., Tokyo, Japan; accuracy ± 0.1 kg) and a wall-mounted stadiometer (SECA Ltd., Hamburg, Germany; accuracy ± 0.1 cm), respectively. Following anthropometric assessment, players completed a standardized warm-up consisting of 3 min of low-intensity running, 3 min of dynamic stretching, and 3 min of moderate-to-high intensity movements. After the warm-up, participants undertook the following physical performance tests, all performed in the same order by every player: (1) two trials of a 30-m linear sprint, with split times recorded at 5 and 30 m to evaluate acceleration and maximum sprint speed; (2) two trials of the Modified Agility Test (MAT), adapted to preserve a constant forward running direction [23], based on the original protocol by Sassi et al. (2009), to assess change-of-direction ability [24]; (3) two trials of a Dribbling test, with the same structure of the MAT but dribbling the ball [23] and (4) one trial of the 30–15 Intermittent Fitness Test to assess intermittent aerobic endurance, administered according to the standardized procedures described by Buchheit et al. (2008) [25]. All assessments were performed on artificial turf, with participants wearing sport-specific footwear (studded boots) and training apparel. Sprint, agility and dribbling times (5 m, 30 m, MAT and Dribbling) were recorded using a set of photoelectric timing gates (Witty System, Microgate, Bolzano, Italy). A two-minute passive recovery period was allowed between sprint and agility trials to ensure maximal performance in each attempt. Players were verbally encouraged throughout the testing battery to achieve maximal effort.

#### 2.3.2. Physical Requirements in Matches and Training Sessions

The physical demands during both matches and training sessions were monitored using a wearable device (OLI, Oliver IMU^®^, Barcelona, Spain), which integrates a GPS module sampling at 10 Hz and an inertial measurement unit (IMU; accelerometer and gyroscope) sampling at 50 Hz. The devices were mounted on the dominant leg of each outfield player using a specific holder designed as a calf sleeve, which included a pocket to secure the device firmly without perturbations or displacement. The device was vertically aligned with the leg, positioned at the level of the gastrocnemius muscle belly. Devices were initialized in a static position before use, then placed in the holder and worn throughout the activity. At the end of each session, the devices were removed, powered off in a static position, and subsequently connected to the proprietary software to download and process the data. All procedures were carried out in accordance with the manufacturer’s instructions. The OLIVER system has shown validity and reliability for measuring external load variables in football contexts [26,27], and it has also been previously employed in CP football with comparable samples [28,29]. Additionally, all distance-related metrics were normalized to actual playing time and expressed as values per minute to enable standardized comparisons.

- Maximal velocity: The highest movement speed reached by the player during the match/training session.
- Total distance: The cumulative distance covered by the player throughout the match/training session.
- Walking distance: Distance covered at a walking pace (<6 km·h^−1^).
- Low-intensity (LI) running: Distance covered at speeds between 6–12 km·h^−1^.
- Moderate-intensity (MI) running: Distance covered at speeds between 12–18 km·h^−1^.
- High-intensity (HI) running: Distance covered at high speeds (>18 km·h^−1^).
- Dribbling distance: Total distance covered while maintaining control of the ball through dribbling.
- Moderate-intensity (MI) accelerations: Distance covered while accelerating between 2 and 3 m·s^−2^.
- High-intensity (HI) accelerations: Distance covered while accelerating beyond 3 m·s^−2^.
- Moderate-intensity (MI) decelerations: Distance covered while decelerating between −2 and −3 m·s^−2^.
- High-intensity (HI) decelerations: Distance covered while decelerating beyond −3 m·s^−2^.

### 2.4. Statistical Analysis

Data are presented as means and standard deviations. To illustrate the physical requirements of training sessions and matches in chronological order, raw data were used. For statistical analyses, distance-related variables were normalized to actual playing time. Each physical data point from training or match play was treated as an independent observation, as the data were obtained from distinct sessions or matches, each characterized by different tactical approaches, training objectives, match outcomes, or levels of opposition, thus constituting unique conditions. The final dataset comprised 60 match observations and 96 training observations. Post hoc power analyses were conducted for the primary statistical tests. Both the independent *t*-test (training [n = 96] vs. match [n = 60]; α = 0.05) and the repeated-measures ANOVA (1 × 5 within-subject design; n = 12, α = 0.05, assuming a within-subject correlation of r = 0.80) achieved statistical power above 0.80 for detecting effects of moderate magnitude, indicating that the study was adequately powered for its main comparisons. An independent samples *t*-test was conducted to examine potential differences in the physical requirements of players between training sessions and competitive matches. Additionally, two one-way ANOVAs were performed, each followed by Bonferroni post hoc tests, to assess possible differences in physical requirements across individual matches and training sessions. Effect sizes (ES) and their 95% confidence intervals (CI) were calculated using Cohen’s d, and interpreted as follows: large (≥0.8), moderate (0.5–0.79), small (0.2–0.49), and trivial (<0.2) [30]. The relationships between field-based physical performance test outcomes and physical requirements during both training and match play were analyzed using Pearson’s correlation coefficients (r), interpreted according to Hopkins et al. (2009) as: trivial (<0.10), small (0.10–0.29), moderate (0.30–0.49), high (0.50–0.69), very high (0.70–0.89), and almost perfect (>0.90) [31]. All data were analyzed using Microsoft Excel 2016 (Microsoft, Seattle, WA, USA) and JASP software (version 0.19.1.0, Amsterdam, The Netherlands). Statistical significance was set at *p* < 0.05.

## 3. Results

**Figure 1:**
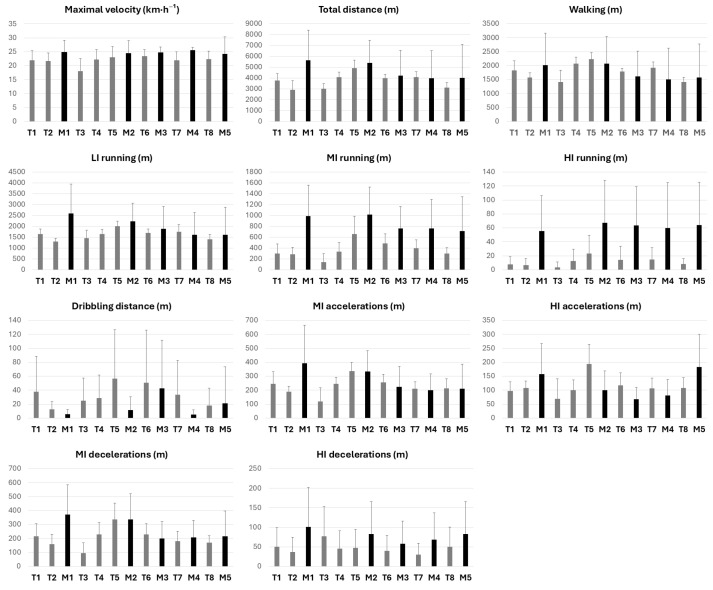
Chronological representation of players’ physical requirements across training sessions and matches throughout the tournament (mean ± standard deviation). The schedule included one rest day after Match 2, at the end of the group stage, while all other days involved either a match or a training session.

Descriptive statistics (means and standard deviations) of players’ physical requirements during training sessions and matches are presented chronologically in Figure 1. A significant difference was observed in players’ total practice time between training sessions and matches (t = 4.37, *p* < 0.01, ES(d) = 0.79, moderate difference), and therefore all distance-related variables were normalized to playing time for the subsequent statistical analyses.

Maximal velocity also differed between training and match conditions (t = 4.54, *p* < 0.01, ES(d) = 0.82, large difference), with higher values in competition. Once normalized by playing time, total distance covered per minute, as well as distances covered at moderate and high intensity, were higher in competition in comparison to training sessions: Total distance per minute (t = 2.79, *p* = 0.01, ES(d) = 0.50, moderate difference), MI running (t = 7.27, *p* < 0.01, ES(d) = 1.31, large difference), and HI running (t = 8.06, *p* < 0.01, ES(d) = 1.45, large difference). In contrast, no significant differences were found between training sessions and matches for distances covered at low intensity (t = 0.44–1.81, *p* = 0.07–0.66, ES(d) = 0.08–0.33, trivial to small differences). No differences were also observed in the distances covered during moderate- or high-intensity accelerations and decelerations (t = 0.22–1.60, *p* = 0.11–0.83, ES(d) = 0.04–0.23, trivial to small differences).

Table 1 displays the means and standard deviations for each variable during matches. No significant differences were found between World Championship matches for any of the analyzed variables (F = 0.17–0.88; *p* = 0.49–0.95). Maximal velocity, MI running and HI running showed significant differences across training sessions during the tournament (F = 2.08–2.76, *p* = 0.05–0.01). Means, standard deviations, and pairwise comparisons for training sessions are presented in Table 2.

Significant relationships were identified between physical performance tests and the physical requirements observed during official matches (Table 3). Specifically, the MAT showed consistent associations with several competition variables, particularly those related to distance covered at different intensities and mechanical demands. Dribbling performance was linked to a broad set of physical indicators during match play, including both locomotor and acceleration-related metrics. Furthermore, vIFT was associated with certain variables related to high-intensity efforts in competition. No meaningful correlations were observed between sprint test performance and in-match physical demands, except for maximal velocity.

Significant correlations were also observed between several physical performance field tests and the physical requirements recorded during training sessions (Table 4). Notably, performance in the MAT showed associations with multiple locomotor and acceleration-based variables. In addition, dribbling performance was positively related to a wide range of training load indicators, particularly those related to distance covered at different intensities and mechanical actions such as accelerations and decelerations. Moreover, 30-m sprint and vIFT also displayed significant correlations with maximal velocity.

## 4. Discussion

The primary aim of this study was to describe and analyze the physical requirements faced by international CP football players during matches and training sessions conducted throughout an official international competition (2024 IFCPF World Cup). As a secondary aim, the study explored the relationship between performance in standardized field-based physical tests and the physical requirements recorded during both training and competition contexts. The main findings indicate that official matches impose greater physical requirements than interspersed training sessions, particularly in terms of distance covered per minute, peak velocity, and movement at moderate to high intensities. Furthermore, meaningful correlations were observed between performance in physical tests and physical requirements during training and competition.

The results revealed substantial differences between the physical requirements of training and competition in CP football. During matches, players achieved significantly higher peak velocities and covered more distance per minute, particularly at moderate (12–18 km/h) and high (>18 km/h) intensity ranges. These findings align with previous evidence in mainstream football, which consistently shows that match play imposes greater locomotor and physiological demands than training sessions [15,16,17,18]. In able-bodied contexts, players typically cover higher distances per minute during matches than those achieved during training sessions [32]. Moreover, the high-speed running represents up to 16% of the total match distance, with significantly greater distance covered at high and very high-speed running observed in competition compared to training [15,33]. This disparity can be attributed to match-specific contextual factors: the cognitive and emotional intensity of real-game situations increases psychophysiological activation, which in turn promotes more frequent and intense explosive actions; tactical decisions by the opposition apply continuous physical stress requiring reactive responses; and the unpredictable, chaotic nature of competitive play generates unplanned high-intensity efforts [34,35]. Importantly, training drills—particularly small-sided games (SSGs)—often fail to replicate these peak intensities, especially regarding sprint exposure and HI running [36,37,38]. Furthermore, the considerable variability in peak match performance underscores the importance of tailoring training to reflect the specific physical requirements of actual competition. In this regard, the higher maximum velocity observed during matches appears to be associated with game-breaking situations such as counterattacks, emergency defensive recoveries, or runs into space, which are difficult to replicate with the same spontaneity or intensity in training environments [16,39,40]. Despite efforts by the coaching staff to design high-intensity, sport-specific drills, the training sessions held during the tournament did not match the locomotor loads experienced during matches. This aligns with the rationale that, during congested competition periods, training should strike a balance between exposing players to tasks that partially approach match demands and simultaneously facilitating recovery and tactical preparation to ensure readiness for subsequent games. This balanced approach highlights the challenge for practitioners of combining competitive representativeness [41] with recovery-oriented objectives, particularly in teams with limited squad sizes.

A particularly noteworthy finding was the remarkable consistency in physical requirements across matches, with no significant differences observed in any analyzed variables, including those related to high-intensity efforts. These results align with evidence from professional leagues—such as a study on a Portuguese second-tier team—where coefficients of variation for total distance per minute were around 6–7%, and variability in high-speed running and maximal speed were minimal, indicating stable external loads from one match to another [42]. Similarly, in CP football, recent research conducted during the 2024 IFCPF World Cup showed similar values in in-match physical requirements across the five matches of a team during the tournament [9]. This consistency likely reflects tactical coherence within the team, with clearly defined roles and uniform execution of the playing model. Physiologically, the low variability—particularly in high-intensity metrics—suggests that players maintained peak physical performance throughout the tournament, demonstrating effective tolerance to sequences of matches with limited recovery time and accumulated load [42,43]. Nevertheless, some variability can still be observed between matches, as reflected in the greater distances covered in Matches 2 and 4 compared to the others. Although these differences did not reach statistical significance, they may be explained by contextual factors: specifically, these two matches were played against higher-level opponents, which required more defensive work and frequent recovery runs without the ball, potentially resulting in higher total distances covered. These results from mainstream football further support this interpretation by showing that players sustained physical outputs across all matches without decline, despite the congested competition schedule. These results suggest that, at least in this cohort of players, the combination of competitive exposure, interspersed training sessions, and the associated recovery periods did not result in any appreciable decline in physical performance between matches. Consequently, it supports the notion that CP football players, despite neuromotor impairments, can respond effectively to the requirements imposed by the tournament structure employed at the IFCPF World Cup, which involves matches every 48–72 h with planned training sessions in between. This finding is particularly valuable as it provides evidence supporting the feasibility of high-intensity competitive formats in para-football, and the capacity of these athletes to maintain physical performance across physiologically demanding tournaments.

In contrast, training sessions exhibited greater variability in physical requirements, especially in maximum velocity achieved and MI and HI running, with significant differences observed between specific sessions. This variability is well documented in both youth and professional mainstream football contexts, where training days often differ in purpose—ranging from recovery and tactical sessions to physically demanding, high-intensity days—leading to distinct external load profiles. Research in sub-elite youth players (U15–U19) and professional squads has shown moderate to large day-to-day differences in total distance and sprint exposure, with coefficients of variation reaching up to 80% in high-speed running and sprint metrics across the training week [44,45,46]. Such fluctuations are particularly evident in metrics like maximum velocity, which vary depending on session structure and objectives (e.g., small- vs. large-sided games or game-based vs. technical drills) [47,48]. This flexible, needs-based approach reflects intentional periodization, where variability is not only expected but desirable. Furthermore, the significant drop in load observed on the day prior to matches (MD–1) in the present study aligns with established tapering strategies. These involve a progressive reduction in training volume while maintaining or even slightly increasing intensity to optimize readiness and minimize fatigue before competition [45,49]. Similarly, the day following a match (MD+1) is typically structured to promote active recovery, with very low external loads and reduced intensities, particularly in metrics such as high-speed running, sprinting, and acceleration counts. This deliberate reduction facilitates physiological recovery, reduces neuromuscular strain, and helps mitigate cumulative fatigue in congested schedules [50]. Tapering and recovery-focused sessions have been widely adopted in team sports, including football, as effective strategies to enhance performance and reduce injury risk during dense competition periods [51]. Thus, the variability observed across training sessions in this cohort appears to be a positive indicator of strategic planning, balancing overload and recovery to ensure players are physically prepared for competition while mitigating fatigue and injury risk. All these results should, however, be interpreted with caution given the relatively small sample size and the fact that all data were collected from a single national squad. While this provides ecological validity by capturing the demands of an official World Cup, it inevitably constrains the external validity of the findings, which may not generalize to other CP football populations or competition contexts.

Regarding the relationship between player’s physical performance evaluated throughout field-based physical tests and the physical requirements shown in competition and training sessions, the present findings indicate that MAT and dribbling tests exhibit the most consistent and meaningful associations with physical requirements experienced during both competition and training. These results support their ecological validity for assessing functional performance in contexts characterized by frequent changes of direction, reactive movements, and neuromotor constraints. Specifically, the Modified Agility Test (MAT) demonstrated significant correlations with several metrics related to distance covered at varying intensities and acceleration/deceleration demands, particularly during training sessions. These findings support the utility of the MAT as a relevant indicator of an athlete’s ability to cope with the dynamic, multidirectional demands characteristic of gameplay—aligning with previous research that has identified agility as a key performance determinant in intermittent sports contexts [52]. However, in contrast to the findings reported by Peña-González et al. (2025) [9], no significant associations were observed between MAT performance and in-match physical requirements in the present study. In that study, the sample included 1 FT1, 7 FT2, and 3 FT3 players, which may be a key factor for interpreting their results and could partly explain variations observed be-tween studies. This discrepancy may also be attributed to the influence of individual functional impairments among players with CP, the potential mismatch between the pre-planned nature of the MAT and the reactive, perceptual demands of in-match movement behavior, and the modulating effect of tactical roles and positional responsibilities on physical output during competition.

The dribbling test was the only assessment to show multiple significant correlations with players’ physical requirements across training and match contexts, underscoring its value as a comprehensive performance indicator. This aligns with previous findings demonstrating the test’s sensitivity to neuromuscular impairments, particularly those affecting coordination and ball control [53,54]. Dribbling is a highly complex task that integrates sprinting, deceleration, directional changes, and technical skill with the ball, making it especially representative of real match demands. As such, more functionally able players could tend to perform better in this test—mirroring their superior match and training physical requirements. Furthermore, this test has proven useful in talent identification, with international-level players outperforming national-level peers in dribbling ability (Peña-González et al., 2021), reinforcing its discriminant validity within classification and performance pathways. This convergence suggests that the dribbling test may act as a proxy for overall match readiness, offering a practical and ecologically valid tool for coaches to assess integrated physical-technical performance in CP football [23,53,54].

In contrast to the strong associations observed for dribbling, both the vIFT and linear sprint tests (5-m and 30-m) showed limited relationships with game-related physical requirements in CP football. The vIFT, commonly used to estimate intermittent endurance and repeated-effort capacity, was only correlated with maximal velocity during training and showed no meaningful associations during match play. These findings contrast with those reported by Peña-González et al. (2025), who observed stronger associations between intermittent endurance and in-match physical demands using the Yo-Yo IR1 test [9]. This discrepancy may reflect contextual differences between studies—such as opposition level, match density, or players’ competitive readiness—which could have modulated the extent to which aerobic and repeated-effort capacities were linked to the players’ physical requirements in competition or training sessions. Rather than challenging the relevance of intermittent endurance per se, our results suggest that its influence on match requirements may vary depending on external and tactical conditions.

Likewise, linear sprint tests demonstrated weak or no correlations with in-game demands, aside from minor associations with peak speed. These results may partly reflect a limited capacity of more isolated and decontextualized assessments—such as linear sprint tests—to capture the specific requirements of CP football. Match performance in this population often relies on short, reactive, and multidirectional efforts that are strongly influenced by tactical context and individual adaptations to neuromotor impairment. In this regard, straight-line sprinting may be less representative of real-game behavior. Notably, even athletes with greater impairments or pronounced inter-limb asymmetries—such as those observed in international players with spastic hemiplegia—do not necessarily exhibit poorer linear sprint performance, likely due to effective compensatory strategies developed over time [55]. However, this does not diminish the value of sprint tests in the assessment of physical performance. Their simplicity, reliability, and sensitivity to change make them useful tools for monitoring athletes’ fitness levels, tracking progress following training interventions, and complementing broader functional assessments. Although linear sprinting showed limited associations with in-match physical demands in the present study, its assessment remains relevant in CP football, as previous research has demonstrated its discriminative capacity between competitive levels and its contribution to the identification of high-potential players [23].

This study is not without limitations. The sample size was relatively small and drawn from a single national team, which limits the generalizability of the findings. However, the fact that the data were collected from international-level athletes during an official World Cup adds ecological validity and relevance to performance settings. The cross-sectional design prevents the establishment of causal relationships between physical fitness and in-game behavior. Longitudinal designs would allow for a better understanding of how improvements in specific physical capacities transfer to match performance over time. While the study explored associations between standard physical tests and physical requirements during training and matches, the influence of contextual variables such as match outcome, tournament phase, player position, or opponent strength was not analyzed, and could help to further interpret variability in physical requirements. The absence of between-class comparisons (FT1, FT2, FT3) also limits the applicability of results across the full functional spectrum of CP football players, as physical outputs may be misinterpreted given the higher functionality typically observed in FT3 players and the greater impairment of FT1 players. However, this limitation is mitigated in the present study since the sample included only one FT1 and one FT3 player, with the majority of participants belonging to the FT2 class, which helps maintain the representativeness of the group average. Similarly, the absence of a positional analysis (defenders, midfielders, attackers) represents another limitation, as positional roles could influence external load demands during competition. Additionally, the use of the OLIVER device on a single (dominant) leg may have implications for the interpretation of results, since asymmetries between limbs could influence some locomotor metrics. This methodological consideration should be taken into account when comparing findings with studies using trunk-mounted devices or bilateral measurements. Future studies should address these limitations by including larger and more diverse samples, adopting multiteam or multicountry designs, and incorporating additional biomechanical, perceptual, or contextual variables to refine performance profiling in this population.

### Practical Applications

The findings of this study can be translated into specific recommendations for coaches and practitioners in CP football. Training tasks should be designed to reproduce competitive demands by incorporating open play, tactical unpredictability, and explosive actions, but always balanced with the need to promote recovery and ensure readiness for the following match, particularly during congested tournament schedules. In terms of load management, training should be periodized around competition, implementing structured tapering strategies in the days prior to matches and organizing active recovery sessions immediately afterward. Regarding physical testing, dribbling and agility assessments should be prioritized in routine monitoring, as they provide the strongest indication of functional readiness and can guide individualized training. Linear sprint and intermittent endurance tests, although showing weaker associations with match demands, should still be included to monitor long-term fitness progression and to differentiate performance levels among players.

## Figures and Tables

**Table 1:** Descriptive data of players’ physical requirements during official World Championship matches.

In-Match Physical Requirements	Match 1	Match 2	Match 3	Match 4	Match 5	Average
Opponent (World Ranking)	Ukraine (1st)	Argentina (7th)	Japan (15th)	Venezuela (13th)	Ireland (8th)	
Phase and result (Spain vs. Opponent)	Group; 0–4	Group; 0–0	9th to 16th; 2–0	9th to 12th; 4–0	9th to 10th; 1–5	
Maximal velocity (k·h^−1^)	24.84 ± 4.24	24.49 ± 4.50	24.71 ± 1.97	25.62 ± 1.01	24.26 ± 6.03	24.78 ± 3.55
Total distance (m·min^−1^)	84.15 ± 21.72	188.33 ± 314.90	83.08 ± 102.39	169.84 ± 324.47	95.85 ± 114.55	124.25 ± 175.61
Walking (m·min^−1^)	29.67 ± 4.60	70.42 ± 121.34	29.76 ± 31.11	70.78 ± 143.86	39.95 ± 51.73	48.12 ± 70.53
LI running (m·min^−1^)	37.17 ± 14.18	86.77 ± 158.97	36.70 ± 47.47	65.72 ± 122.87	37.16 ± 46.04	52.70 ± 77.91
MI running (m·min^−1^)	16.62 ± 10.87	29.89 ± 36.95	15.62 ± 23.67	31.83 ± 56.17	15.92 ± 14.68	21.98 ± 28.47
HI running (m·min^−1^)	0.69 ± 0.59	1.24 ± 0.98	0.76 ± 0.84	1.52 ± 2.01	1.99 ± 2.81	1.24 ± 1.45
Dribbling distance (m·min^−1^)	0.27 ± 0.47	0.96 ± 2.48	0.63 ± 0.87	0.06 ± 0.08	0.07 ± 0.13	0.40 ± 0.81
MI accelerations (m·min^−1^)	5.68 ± 3.01	13.37 ± 26.12	4.05 ± 4.30	6.98 ± 11.85	4.44 ± 5.07	6.90 ± 10.07
HI accelerations (m·min^−1^)	2.31 ± 1.08	3.45 ± 5.98	1.05 ± 0.75	2.86 ± 5.29	21.61 ± 57.75	6.26 ± 14.17
MI decelerations (m·min^−1^)	6.03 ± 3.01	11.06 ± 18.17	4.07 ± 5.59	7.94 ± 14.34	4.98 ± 6.13	6.82 ± 9.45
HI decelerations (m·min^−1^)	3.01 ± 1.66	5.14 ± 8.34	2.09 ± 2.85	4.92 ± 9.64	2.12 ± 1.57	3.46 ± 4.81

LI: low intensity; MI: moderate intensity; HI: high intensity. Spain world ranking: 10th.

**Table 2:** Descriptive data of players’ physical requirements during official World Championship training sessions.

In-Training Physical Requirements	TS1	TS2	TS3	TS4	TS5	TS6	TS7	TS8	Average
Maximal velocity (k·h^−1^)	21.94 ± 3.39	21.63 ± 2.95	18.08 ± 4.46	22.18 ± 3.55	22.98 ± 3.85 ^c^	23.43 ± 2.35 ^c^	21.96 ± 2.99	22.31 ± 2.87	21.81 ± 3.30
Total distance (m·min^−1^)	124.16 ± 158.34	103.26 ± 151.56	98.23 ± 140.24	139.04 ± 198.12	169.54 ± 234.71	138.45 ± 195.54	148.75 ± 215.73	92.44 ± 126.53	126.73 ± 177.60
Walking (m·min^−1^)	56.60 ± 68.82	50.13 ± 68.48	44.89 ± 64.58	65.15 ± 86.49	75.95 ± 105.45	61.21 ± 88.76	66.04 ± 95.40	40.47 ± 54.76	57.56 ± 79.09
LI running (m·min^−1^)	56.69 ± 78.12	47.45 ± 70.20	44.80 ± 57.07	57.37 ± 83.08	68.75 ± 98.52	58.55 ± 82.33	65.07 ± 94.41	43.39 ± 61.99	55.26 ± 78.22
MI running (m·min^−1^)	10.74 ± 12.86	9.66 ± 11.66	8.19 ± 18.38	15.78 ± 27.11	24.03 ± 30.66 ^b,c^	18.18 ± 24.29	17.11 ± 25.80	8.44 ± 10.10 ^e^	14.02 ± 20.11
HI running (m·min^−1^)	0.13 ± 0.15	0.24 ± 0.41	0.38 ± 1.07	0.75 ± 1.67 ^a^	0.81 ± 1.32 ^a^	0.51 ± 0.82 ^a^	0.53 ± 0.64 ^a^	0.14 ± 0.15 ^d,e,f,g^	0.44 ± 0.78
Dribbling distance (m·min^−1^)	2.60 ± 6.86	0.37 ± 0.46	0.65 ± 1.70	0.63 ± 0.78	1.02 ± 1.10	0.84 ± 1.01	1.90 ± 4.28	0.40 ± 0.67	1.05 ± 2.11
MI accelerations (m·min^−1^)	6.98 ± 7.24	6.78 ± 9.47	5.28 ± 10.80	9.63 ± 16.04	12.71 ± 19.69	9.80 ± 14.78	8.84 ± 15.03	6.74 ± 10.28	8.35 ± 12.92
HI accelerations (m·min^−1^)	2.91 ± 3.04	3.88 ± 5.82	4.59 ± 10.90	4.08 ± 7.18	7.55 ± 10.00	4.57 ± 7.26	4.49 ± 7.57	3.07 ± 4.44	4.39 ± 7.03
MI decelerations (m·min^−1^)	7.60 ± 9.28	6.35 ± 9.51	3.63 ± 6.45	7.94 ± 11.39	13.65 ± 20.61	8.97 ± 12.69	8.90 ± 15.24	5.76 ± 8.59	7.85 ± 11.72
HI decelerations (m·min^−1^)	3.45 ± 3.49	4.06 ± 6.82	6.02 ± 14.89	4.31 ± 7.20	6.56 ± 9.38	3.30 ± 4.00	4.04 ± 5.65	2.85 ± 3.97	4.32 ± 6.92

TS: training session; LI: low intensity; MI: moderate intensity; HI: high intensity. ^a^ statistical difference with TS1; ^b^ statistical difference with TS2; ^c^ statistical difference with TS3; ^d^ statistical difference with TS4; ^e^ statistical difference with TS5; ^f^ statistical difference with TS6; ^g^ statistical difference with TS7.

**Table 3:** Pearson’s correlation coefficients between in-match physical requirements and physical performance tests.

In-Match Physical Requirements [M ± SD]	5-m Sprint (s)	30-m Sprint (s)	MAT (s)	Dribbling (s)	vIFT (km·h^−1^)
	[1.22 ± 0.06]	[4.14 ± 0.20]	[6.08 ± 0.34]	[8.70 ± 0.53]	[18.27 ± 1.49]
Maximal velocity (k·h^−1^)	−0.38 *	−0.34 *	−0.46 *	−0.46 *	0.12
Total distance (m·min^−1^)	−0.14	−0.09	−0.15	−0.37 *	−0.04
Walking (m·min^−1^)	−0.18	−0.13	−0.14	−0.36 *	−0.08
LI running (m·min^−1^)	−0.13	−0.08	−0.16	−0.36 *	−0.04
MI running (m·min^−1^)	−0.01	0.01	−0.20	−0.40 *	0.09
HI running (m·min^−1^)	−0.22	−0.21	−0.04	−0.17	0.17
Dribbling distance (m·min^−1^)	−0.19	−0.16	−0.03	−0.15	−0.01
MI accelerations (m·min^−1^)	−0.15	−0.12	−0.10	−0.31	−0.03
HI accelerations (m·min^−1^)	−0.18	−0.13	−0.06	−0.24	−0.07
MI decelerations (m·min^−1^)	−0.12	−0.09	−0.14	−0.39 *	0.03
HI decelerations (m·min^−1^)	−0.09	−0.06	−0.15	−0.38 *	−0.03

LI: low intensity; MI: moderate intensity; HI: high intensity; * *p* < 0.05.

**Table 4:** Pearson’s correlation coefficients between in-training physical requirements and physical performance tests.

In-Training Physical Requirements	**5-m** Sprint (s)	**30-m** Sprint (s)	MAT (s)	Dribbling (s)	vIFT (km·h^−1^)
[M ± SD]	[1.22 ± 0.06]	[4.14 ± 0.20]	[6.08 ± 0.34]	[8.70 ± 0.53]	[18.27 ± 1.49]
Maximal velocity (k·h^−1^)	−0.17	−0.29 *	−0.27 *	−0.05	0.24 *
Total distance (m·min^−1^)	−0.09	−0.01	−0.31 *	−0.64 *	0.07
Walking (m·min^−1^)	−0.10	−0.02	−0.30 *	−0.64 *	0.08
LI running (m·min^−1^)	−0.10	−0.01	−0.31 *	−0.65 *	0.07
MI running (m·min^−1^)	−0.03	−0.09	−0.35 *	−0.64 *	0.00
HI running (m·min^−1^)	−0.25	−0.21	−0.07	−0.37 *	0.03
Dribbling distance (m·min^−1^)	−0.17	−0.11	−0.09	−0.33 *	0.10
MI accelerations (m·min^−1^)	−0.12	−0.05	−0.27 *	−0.60 *	0.07
HI accelerations (m·min^−1^)	−0.16	−0.08	−0.24	−0.57 *	0.07
MI decelerations (m·min^−1^)	−0.05	−0.02	−0.32 *	−0.63 *	0.04
HI decelerations (m·min^−1^)	−0.13	−0.06	−0.25	−0.56 *	0.08

LI: low intensity; MI: moderate intensity; HI: high intensity; * *p* < 0.05.

## Data Availability

The original contributions presented in the study are included in the article, further inquiries can be directed to the corresponding author.
